# Supplementary material for: Ascending dorsal column sensory neurons respond to spinal cord injury and downregulate genes related to lipid metabolism
Source: Sci Rep. 2021 Jan 11;11:374. doi: 10.1038/s41598-020-79624-0 (PMC7801468; doi:10.1038/s41598-020-79624-0)
Supplement: Supplementary file 1 — Supplementary Information. [file 41598_2020_79624_MOESM1_ESM.pdf]

Supplementary information

Ascending dorsal column sensory neurons respond to spinal cord injury and downregulate genes related to lipid metabolism

Eric E. Ewan[1], Oshri Avraham[1], Dan Carlin[1], Tassia Mangetti Goncalves[1], Guoyan Zhao[1], and Valeria Cavalli[1,2,3*]

1. Department of Neuroscience, Washington University School of Medicine, St Louis 63110, Missouri, USA

2. Hope Center for Neurological Disorders, Washington University School of Medicine, St. Louis, Missouri 63110, USA

3. Center of Regenerative Medicine, Washington University School of Medicine, St. Louis, Missouri 63110, USA

*Correspondence to: Valeria Cavalli, Department of Neuroscience, Washington University School of Medicine, Campus Box 8108, 660 S. Euclid Ave, St. Louis, MO 63110-1093 Phone: 314 362 3540, Fax: 314 362 3446, E-mail: cavalli@wustl.edu

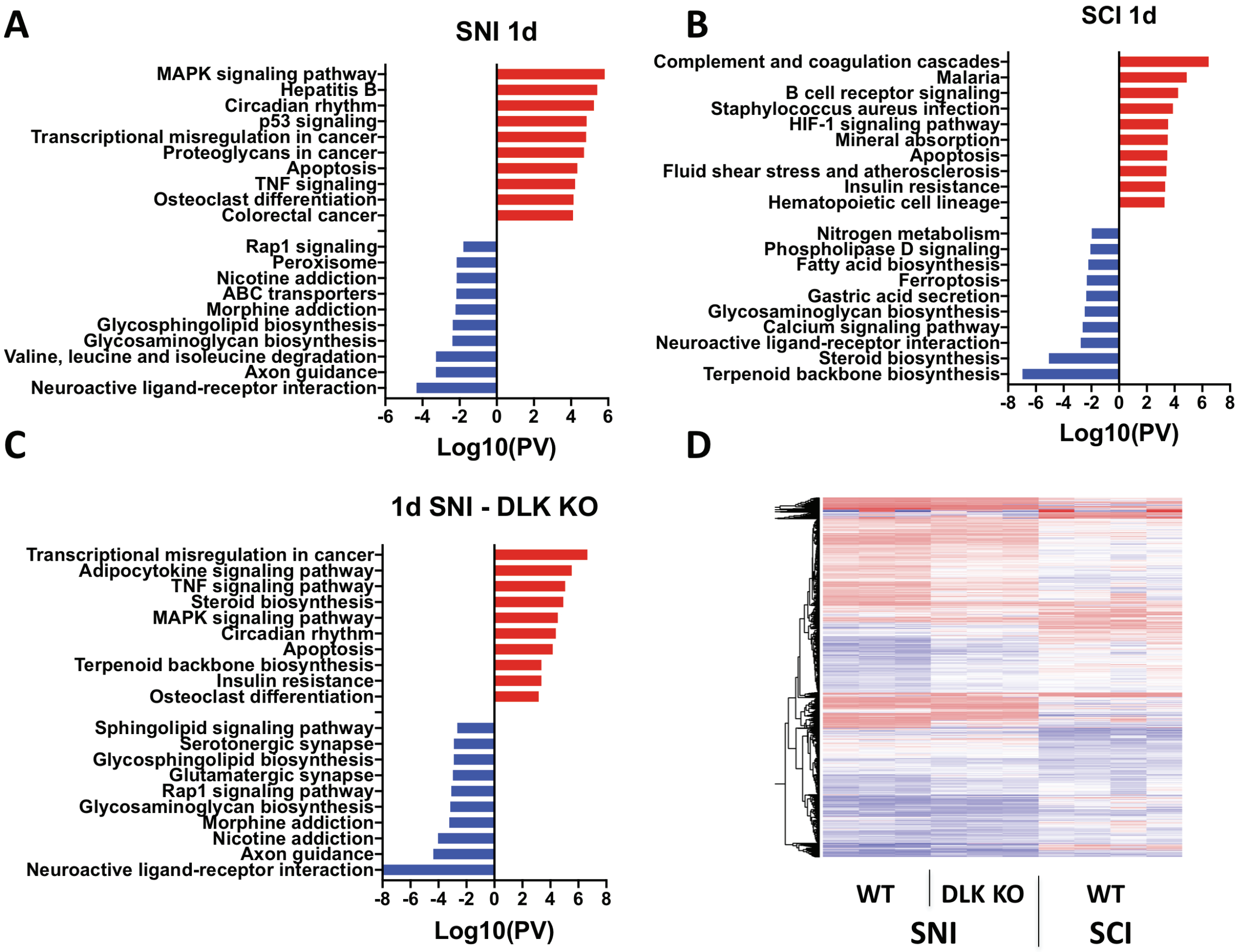

Figure S1. Pathway analysis and transcriptional comparison after sciatic nerve injury (SNI) and spinal cord injury (SCI) in dorsal root ganglion (DRG). A-C) Pathway analysis for the most significantly enriched pathways associated with upregulated (red) and downregulated (blue) differentially expressed (DE) genes after SNI (n=3 mice) or SCI (n=4 mice) in wildtype or SNI in dual leucine zipper kinase (DLK) knockout mice (n=3 mice; p-adj < 0.1; KEGG 2016). **D**) Heatmap of all upregulated (red) and downregulated (blue) DE genes for each subject and each condition.

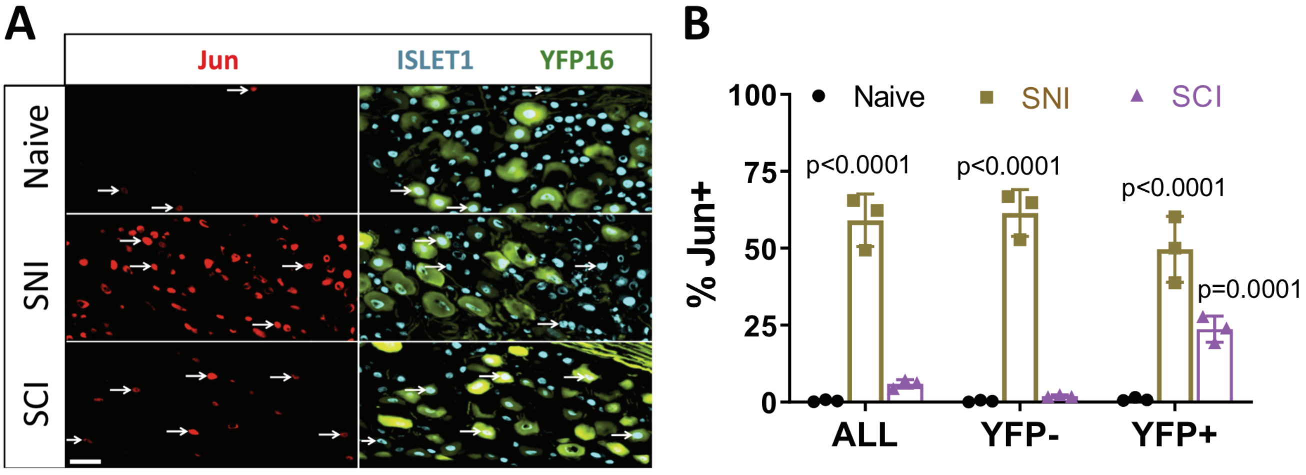

Figure S2. Jun upregulation is specific to dorsal column (DC) neurons after spinal cord injury (SCI). A) Representative images of L4 dorsal root ganglion (DRG) neurons labeled with Jun and Islet1 antibodies in Thy1YFP16 mice in naive or 3 days after sciatic nerve injury (SNI) or SCI. **B**) Quantification of **A** indicating percentage of Jun positive, Islet-1 labeled neuronal nuclei in all neurons, as well as YFP negative and YFP positive neurons, for each condition (n=3 mice/group; 2-way ANOVA). White arrowheads point to Jun positive neuronal nuclei. *$p < 0.05$, ***$p < 0.001$

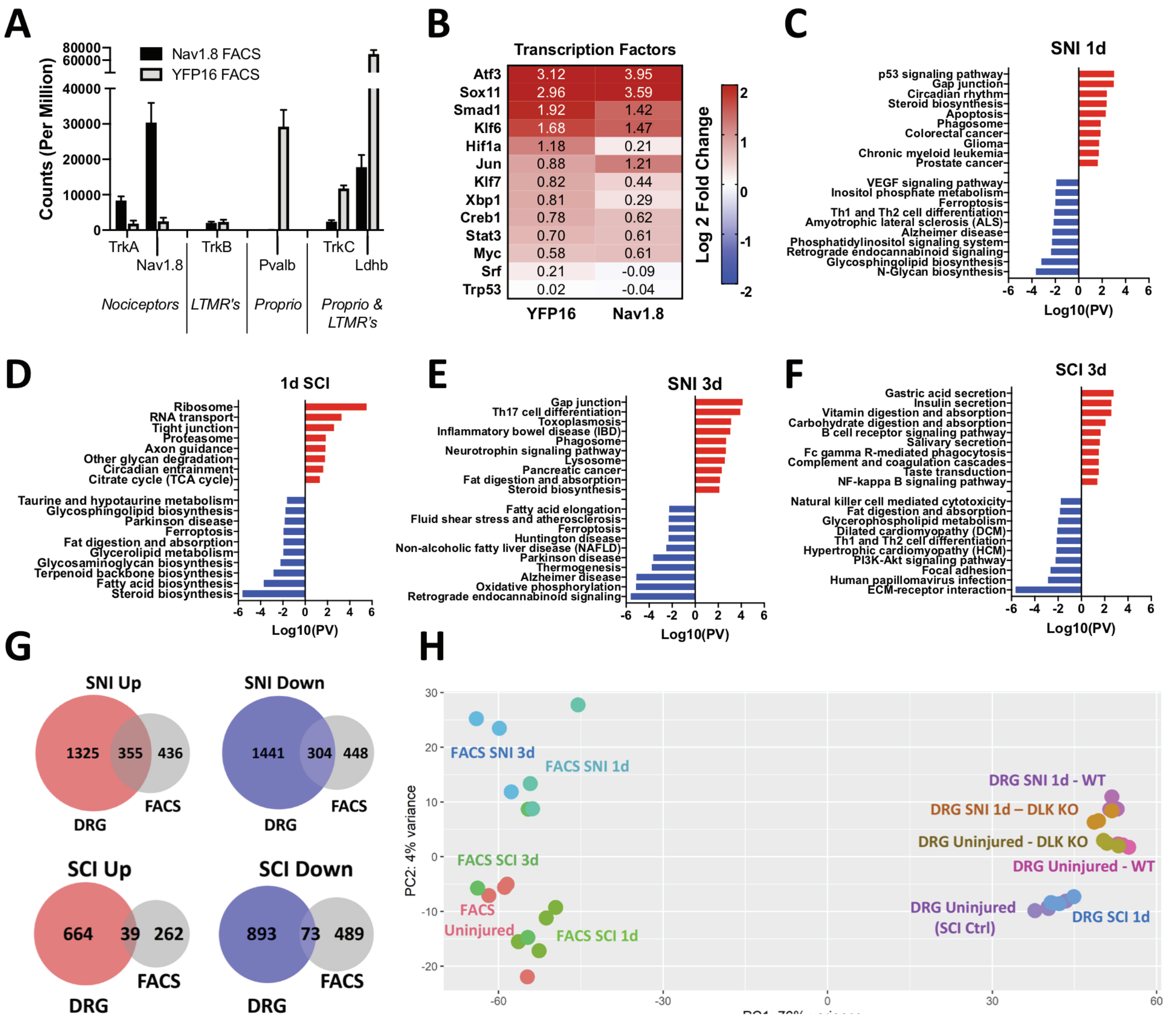

Figure S3. Pathway analysis and transcriptional response of DC neurons 1 and 3 days after sciatic nerve injury (SNI) and spinal cord injury (SCI).

A) Comparison of total counts of genes associated with three predominant neuronal subtypes in the DRG (nociceptors, low-threshold mechanoreceptors (LTMR's), and proprioceptors (Proprio)) between fluorescence-activated cell sorted (FACS) nociceptors (Nav1.8) and DC neurons (Thy1YFP16). **B**) Heatmap of known regeneration-associated transcription factors (RATF's) 3 days after SNI in FACS sorted nociceptors (Nav1.8) and DC neurons (Thy1YFP16). **C-F**) Pathway analysis for the most significantly enriched pathways associated with upregulated (red) and downregulated (blue) differentially expressed (DE) genes 1 and 3 days after SNI (n=3 mice) or SCI (n=4 mice (1d), n=3 mice (3d)) in DC neurons (KEGG 2016). **G-H**) Proportional venn diagrams for DE genes upregulated (red) or downregulated (blue) 1 day after SNI and SCI in whole DRG (p-adj<0.1) compared with DC neurons (p-adj<0.1, RUvR = 2).

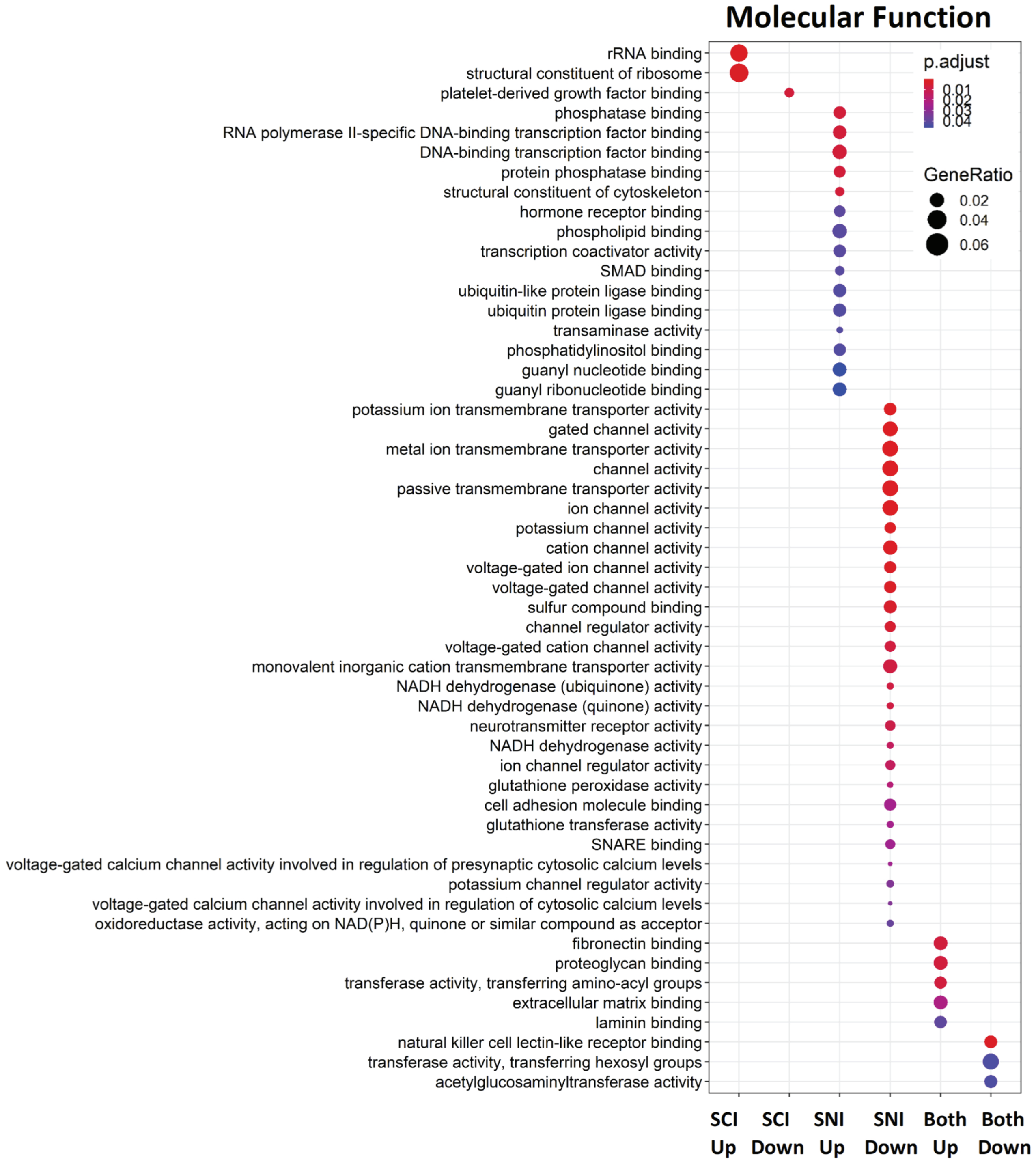

Figure S4. GO analysis of genes containing and ATF3 binding motif after SNI or SCI

Gene Ontology (GO) enrichment analysis was performed on differentially expressed (DE) genes that have ATF3 binding sites in the promoter upstream sequences. DE genes from 1d and 3d post injured were combined in each injury condition. The DE genes were categorized into genes that were uniquely upregulated in SCI or SNI, uniquely downregulated in SCI or SNI as well as genes that were up- or down-regulated in both SCI and SNI. The graph shows the GO terms in the Molecular Function category enriched in each gene list.

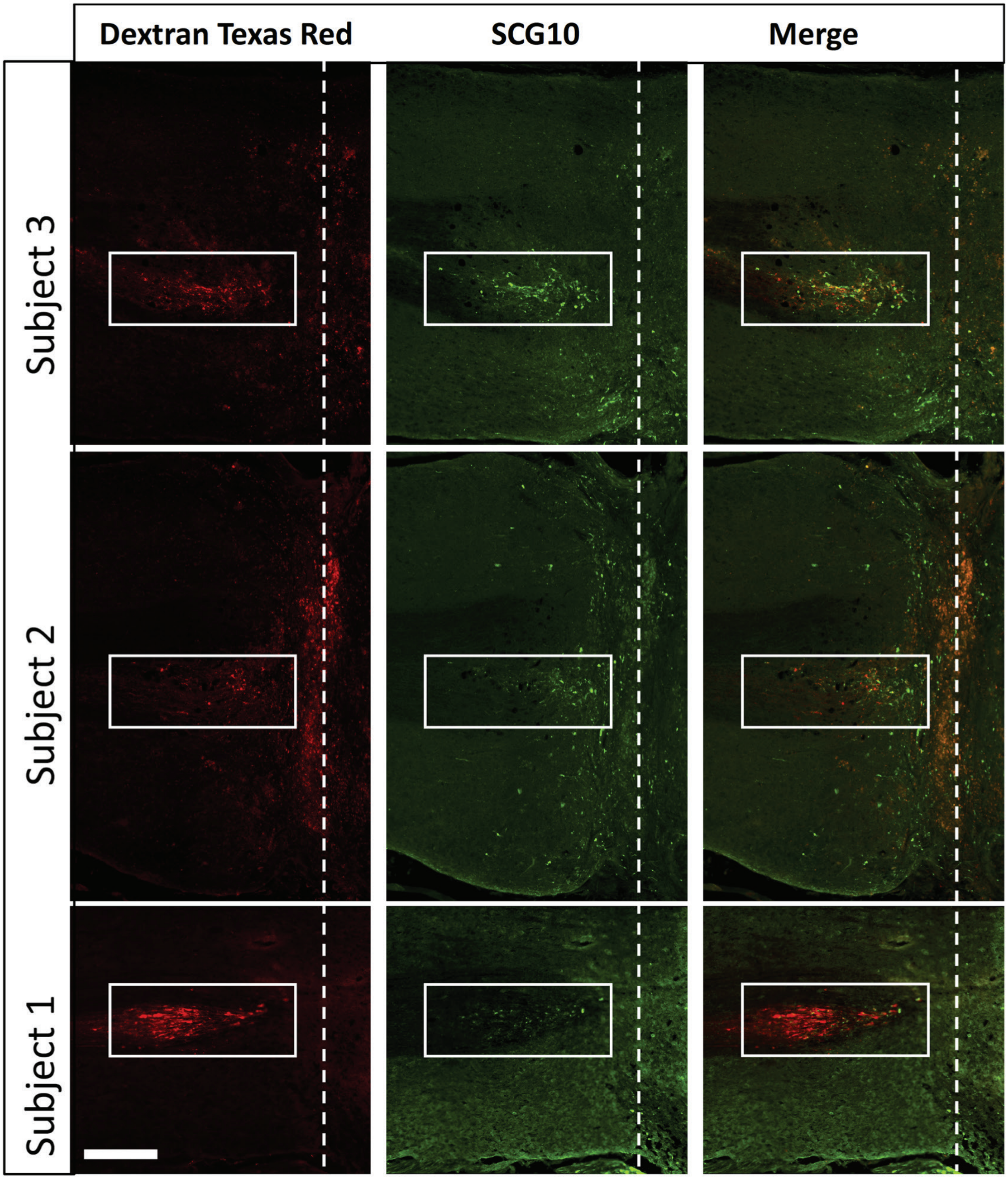

Figure S5. Conditioning nerve injury leads to accumulation of SCG10 in injured ascending sensory axons.

Horizontal sections of the dorsal column labeled with SCG10 and dextran-labeled conditioned axons that was injected into the ipsilateral sciatic nerve. Representative horizontal spinal images are shown for 3 different subjects.

Supplementary Tables

Table 1. List of genes significantly upregulated after SCI (green) or SNI in wildtype (gray) and DLK KO (blue) mice 1d after injury in whole DRG.

Table 2. List of genes significantly downregulated after SCI (green) or SNI in wildtype (gray) and DLK KO (blue) mice 1d after injury in whole DRG.

Table 3. List of all genes after SCI (green) or SNI in wildtype (gray) and DLK KO (blue) mice 1d after injury in whole DRG.

Table 4. List of genes that were inversely expressed between SNI and SCI 1d after injury in whole DRG.

Table 5. List of all genes after SCI (green) or SNI in mice 1d (lighter shade) or 3d (darker shade) after injury in FACS-sorted dorsal column neurons.

Table 6. List of genes significantly upregulated after SCI (green) or SNI in mice 1d (lighter shade) or 3d (darker shade) after injury in FACS-sorted dorsal column neurons.

Table 7. List of genes significantly downregulated after SCI (green) or SNI in mice 1d (lighter shade) or 3d (darker shade) after injury in FACS-sorted dorsal column neurons.

Table 8. List of genes containing ATF3 binding motifs regulated by SNI and SCI
